# Papillomavirus can be transmitted through the blood and produce infections in blood recipients: Evidence from two animal models

**DOI:** 10.1080/22221751.2019.1637072

**Published:** 2019-07-25

**Authors:** Nancy M. Cladel, Pengfei Jiang, Jingwei J. Li, Xuwen Peng, Timothy K. Cooper, Vladimir Majerciak, Karla K. Balogh, Thomas J. Meyer, Sarah A. Brendle, Lynn R. Budgeon, Debra A. Shearer, Regina Munden, Maggie Cam, Raghavan Vallur, Neil D. Christensen, Zhi-Ming Zheng, Jiafen Hu

**Affiliations:** a The Jake Gittlen Laboratories for Cancer Research, Pennsylvania State University College of Medicine, Hershey, PA, USA; b Department of Pathology, Pennsylvania State University College of Medicine, Hershey, PA, USA; c Tumor Virus RNA Biology Section, RNA Biology Laboratory, National Cancer Institute, NIH, Frederick, MD, USA; d Department of Immunology and Microbiology, School of Basic Medical Sciences, Wenzhou Medical University, Wenzhou, People’s Republic of China; e Department of Comparative Medicine, Pennsylvania State University College of Medicine, Hershey, PA , USA; f Integrated Research Facility at Fort Detrick, National Institute of Allergy and Infectious Diseases, NIH, Frederick, MD, USA; g CCR Collaborative Bioinformatics Resource (CCBR), Center for Cancer Research, NCI, NIH, Bethesda, MD, USA; h Advanced Biomedical Computational Science, Frederick National Laboratory for Cancer Research, Frederick, MD, USA; i Department of Microbiology and Immunology, Pennsylvania State University College of Medicine, Hershey, PA, USA

**Keywords:** Papillomavirus, blood transfusion, animal models, CRPV/rabbit model, MmuPV1/mouse model, viral infection

## Abstract

Human papillomaviruses (HPV) contribute to most cervical cancers and are considered to be sexually transmitted. However, papillomaviruses are often found in cancers of internal organs, including the stomach, raising the question as to how the viruses gain access to these sites. A possible connection between blood transfusion and HPV-associated disease has not received much attention. Here we show, in rabbit and mouse models, that blood infected with papillomavirus yields infections at permissive sites with detectable viral DNA, RNA transcripts, and protein products. The rabbit skin tumours induced via blood infection displayed decreased expression of SLN, TAC1, MYH8, PGAM2, and APOBEC2 and increased expression of SDRC7, KRT16, S100A9, IL36G, and FABP9, as seen in tumours induced by local infections. Furthermore, we demonstrate that blood from infected mice can transmit the infection to uninfected animals. Finally, we demonstrate the presence of papillomavirus infections and virus-induced hyperplasia in the stomach tissues of animals infected via the blood. These results indicate that blood transmission could be another route for papillomavirus infection, implying that the human blood supply, which is not screened for papillomaviruses, could be a potential source of HPV infection as well as subsequent cancers in tissues not normally associated with the viruses.

## Introduction

This study grew out of an observation made in 2005 [1] that a subset of children with HIV also had detectable levels of human papillomavirus (HPV) in their blood. Some of these children were hemophiliacs who had contracted HIV through contaminated transfusions. HPV has been detected in malignant tissues, including those of the head and neck [2], esophagus [3], lung [4], colorectum [5,6], prostate [7,8], breast [9,10] and stomach [11,12]. We asked: (1) Could blood be a non-sexual mode for the transmission of papillomavirus infections? (2) Is the public at risk since the blood supply is not screened for papillomaviruses?

HPV is strictly species-specific [13] and thus, cannot be studied directly in animals. Our laboratory has two preclinical animal models with their own naturally occurring papillomaviruses [14–17]. We have developed methods that allow us to use these models to test the possibility of papillomavirus transmission by blood.

The Cottontail Rabbit Papillomavirus (CRPV) infection model is a gold standard preclinical model for HPV-associated infections and diseases [14,18]. This model has been in use in our laboratory for more than three decades. CRPV infections produce cutaneous tumours which, in time, progress to cancer [15]. Using this model, (1) We detected viral DNA in the blood of animals infected with CRPV; (2) We produced papillomavirus infections by injecting virus and viral DNA into the ear vein; and (3) We transfused blood containing CRPV virus from a previously transfused animal to an uninfected rabbit and demonstrated papilloma formation. These results showed that blood containing papillomavirus or viral DNA could yield infections in recipients and *strengthened the possibility that human papillomaviruses can be transmitted by blood.*

We then extended these studies to the novel mouse papillomavirus (MmuPV1) model. MmuPV1 is the first mouse papillomavirus suitable for large-scale laboratory studies. MmuPV1 has both cutaneous and mucosal tropisms [19–21]. For this study, (1) Naïve animals were infected with virus via the tail vein. As for the rabbit, infections developed at susceptible sites. Furthermore *infections were detected in the stomachs of three animals*; (2) Blood was drawn from infected mice and transfused into naïve animals. Infections developed at both cutaneous and mucosal sites in these animals. In addition, an *infection was detected in the stomach of one of the six animals*.

Taken together, these results provide direct evidence of blood transmission of papillomaviruses. The findings call into question the safety of the human blood supply and suggest that consideration be given to the screening of blood for HPV contamination.

## Materials and methods

### Animals and viral infections

All animal work was approved by the Institutional Animal Care and Use Committee of Pennsylvania State University’s College of Medicine (COM) and all procedures were performed in accordance with the required guidelines and regulations. Outbred New Zealand White (NZW) rabbits were purchased from Robinson and housed individually. Male and female Hsd: NU outbred athymic nude mice (Foxn1^nu/nu^) (6–8 weeks old) were obtained from ENVIGO and were housed (2–3 mice/cage) in sterile cages within sterile filter hoods and were fed sterilized food and water in the COM BL2 animal core facility.

### Viral stock

Virus was isolated from cottontail rabbit tumours or tumours on the tails of mice from our previous studies [22,23]. In brief, papillomas from the cottontail rabbits or tumours scraped from mouse tails were homogenized in phosphate-buffered saline (1 × PBS) using a Polytron homogenizer (Brinkman PT10-35) at highest speed for three minutes while chilling in an ice bath. The homogenate was spun at 10,000 rpm and the supernatant was decanted into Eppendorf tubes for storage at −20°C. For these experiments, the virus was diluted 1:5 in 200 μl of 1 × PBS and was passed through a 0.2 μm cellulose acetate sterile syringe filter. Viral DNA extracted from 5 μl of this virus stock was quantitated using qPCR as described [24].

### Titration of CRPV virus and viral DNA

 Viral skin infections can be initiated with either virus [25] or viral DNA cloned into a plasmid vector [26,27]. Our earlier work demonstrated the importance of wounding prior to infection [25]. To identify the optimal concentration for both virus and viral DNA infections in rabbits for the current work, titration studies were conducted using the pre-wounding skin infection method developed in our laboratory [25]. Results demonstrated that more than 2.75 × 10^6^ virion DNA equivalents (Supplementary Table 1) and 1.3 × 10^10^ copies for cloned infectious CRPV DNA (Supplementary Table 4) are required to guarantee 100% tumour appearance at locally infected sites. For this study, 500 µl of virus stock (2.75 × 10^10^ virion DNA equivalents) and 500 µg of the cloned CRPV DNA (estimated to be 4.6 × 10^11^ copies/µl blood) were used for IV infection to maximize the opportunity to detect disease.

### Routes of infection and sample collection

CRPV infectious virions used in the current study were from a viral stock previously reported [28]. CRPV DNA was cloned into a pUC19 vector as described in our previous publications [29]. Animal back skin sites were pre-wounded three days before infection using our established pre-wounding techniques [25]. For local infections, the rabbits were challenged with either virions or viral DNA at the pre-wounded sites. To investigate whether intravenous (IV) infection with virions (500 µl of the viral stock = 2.75 × 10^10^ viral DNA equivalents) and viral DNA (500 µg of plasmid = 4.6 × 10^11^ copies/µl blood) could induce tumours at distant susceptible back sites, six to eight back skin sites of different groups of rabbits were shaved and pre-wounded with a scalpel blade as previously described [25]. Virus or viral DNA was delivered via the marginal ear vein and then back skin sites were gently re-wounded with a 28G needle [25]. The infected animals were monitored for tumour growth weekly and tumours were measured and documented photographically. Rabbits were euthanized up to 12 weeks after initial viral infection, and tissues were collected for cellular, molecular, and histological analyses. Serum samples were harvested for the detection of anti-viral antibodies. To investigate whether transfusions of blood from an animal that had received virus (500 µl of the viral stock = 2.75 × 10^10^ viral DNA equivalents) by IV injection could transmit infection to naïve siblings, 10 ml of blood was drawn from the injected animal 25 min post IV infection and transfused into naïve animals via the marginal ear vein with pre-wounded back sites. Recipients were monitored for tumour growth and measurements were recorded.

In addition to the CRPV/rabbit model, we used the more recently established MmuPV1/mouse model for a number of our studies [16]. Female mice were subcutaneously inoculated with 3 mg Depo-Provera (Pfizer) in 100 µl PBS three days before the viral infection as previously described [20]. Mice were sedated intraperitoneally with 0.1 ml/10 g body weight with ketamine/xylazine mixture (100 mg/10 mg in 10 ml ddH_2_O). The penile, the lower genital (vaginal) and anal sites were wounded with Doctors’ Brush Picks coated with Conceptrol (Ortho Options, over the counter) as previously described [24]. Tongues were withdrawn using a sterile forceps and microneedles were used to wound the ventral surface of the tongues [24]. Twenty-four hours after wounding, eight female and six male Hsd: Nu athymic mice were again anesthetized and challenged with infectious MmuPV1 virions (1 × 10^8^ viral DNA equivalents) via the tail vein. The injection sites were topically treated with neutralizing antibody (MPV.A4) immediately post injection to neutralize any virions remaining at the injection site. Monitoring was conducted weekly for infection at the muzzle and the tail and progress was documented photographically at these cutaneous sites for each animal [19,20]. Viral infection at three mucosal sites (the vagina or the penis, the anus, and the tongue) was monitored for viral DNA using swabs or by lavage as described previously [24]. At the termination of the experiment, selected organ tissues (kidney, lung, liver, spleen, stomach, and bladder) were harvested to determine whether viral infections could be detected in other unanticipated sites. The tissues were analyzed histologically for the presence of viral DNA and/or L1 and E4 protein production.

To investigate whether blood from actively infected mice could transmit the infection to naïve animals, blood was drawn from one infected male and one infected female mouse and transfused by tail vein injection to three naïve male and female mice respectively. The injection sites were treated topically with neutralizing antibody (MPV.A4) immediately post injection to neutralize any virions remaining at the site. The mice were maintained for up to six months post infection and tissues were collected for cellular, molecular, and histological analyses. Vaginal and anal lavages were conducted using 25 μl of sterile 0.9% NaCl introduced into the vaginal and anal canals with a disposable filter tip. The rinse was gently pipetted in and out of the canals and stored at −20°C before being processed for DNA extraction [24]. For oral lavage, a swab (Puritan Purflock Ultra, Puritan Diagnostics LLC) soaked in 25 μl of sterile 0.9% NaCl was used [20]. For DNA extraction, the DNeasy kit (QIAGEN) was used according to the instructions of the manufacturer. All DNA samples were eluted into 50 µl EB buffer [24].

### RNA isolation from rabbit tumours for quantitative PCR assays

Tumour tissues of NZW rabbits with CRPV infections induced by local skin infection and ear vein IV injections were harvested for qPCR analysis of host genes (Supplementary Table 3). The tissues were homogenized in TriPure reagent (Roche). Total RNA was extracted according to the TriPure extraction protocol and treated with the TURBO DNA-free™ Kit (Ambion) to eliminate all traces of viral DNA. The integrity of RNA were evaluated by an Agilent DNA bioanalyzer and quantified by NanoDrop. Reverse Transcription (RT) was performed with the SuperScript II kit (Thermo Fisher Scientific). 1 µg of total RNA from each tissue was used per reverse transcription reaction to synthesize single-stranded cDNA. cDNA samples were further analyzed using the TaqMan Universal PCR Master Mix (Thermo Fisher) by a StepOne Plus Real-Time PCR System (Applied Biosystems). To avoid interplate variability, differentially expressed gene expression analyses were performed using a single 96-well plate in triplicate. GAPDH was used as an internal control and analyzed in the same plate for each sample. Each threshold cycle (Ct) value of real-time quantitative PCR data from three repeats was individually normalized to GAPDH and analyzed by the 2^−ΔΔCt^ method [30]. All primers and Taqman probes used were listed in Supplementary Table 2.

### RNA-seq analysis

Total RNA isolated using TriPure Reagent (Roche) and the RNeasy Mini kit with on-column DNase-treatment (Qiagen) was used for RNA-seq analysis. The sequencing libraries were constructed from Ribo-minus RNA using TruSeq Stranded Total RNA kit (Illumina RS-122-2201). The obtained libraries were then pooled and sequenced using Illumina TruSeq v4 chemistry, 125-bp paired-end, with 100 million reads depth. The HiSeq RT Analysis software (RTA v1.18.64) was used for base calling. The Illumina bclfastq v2.17 software was used to demultiplex and convert binary base calls and qualities to FASTQ format. The obtained reads were mapped first to the oryCun2.0 (*Oryctolagus cuniculus*) reference genome (https://www.ncbi.nlm.nih.gov/assembly/GCF_000003625.2/) to which had been added a contig containing the cottontail rabbit papillomavirus (CRPV) Hershey strain reference genome (GenBank Acc. No. JF303889.1) permutated at nt 7421. The viral read coverage along the CRPV reference genome was then visualized using the IGV software (http://software.broadinstitute.org/software/igv/). To determine the changes in host gene expression upon viral infection, the reads were remapped to the oryCun2.0 reference genome without the CRPV contig. RNA-seq NGS-datasets were processed using the CCBR Pipeliner utility (https://github.com/CCBR/Pipeliner). Briefly, reads were trimmed of low-quality bases and adapter sequences were removed using Trimmomatic v0.33 [31]. Mapping of reads to the oryCun2.0+CRPV reference genome was performed using STAR v2.5.2b in 2-pass mode [32]. Then, RSEM v1.3.0 was used to quantify gene-level expression, with counts normalized to library size as counts-per-million [32]. Finally, limma-voom v3.34.5 was used for quantile normalization and differential expression [33]. The data discussed in this publication have been deposited in NCBI’s Gene Expression Omnibus [34] and are accessible through GEO Series accession number GSE124211. Genes were considered to be attributed to CRPV infection if they were significantly (adjusted *p* ≤ 0.05) differentially expressed relative to control with absolute fold change relative to control ≥2.0. Genes with ‘unknown’ gene symbols in the oryCun2.0 gene annotation dataset were quantified but excluded from further analysis in this manuscript. Expression data were visualized as heat maps using ClustVis [35].

### Viral DNA copy number analysis

Linearized MmuPV1 genome DNA was used for standard curve determination by Probe qPCR analysis (Brilliant III Ultra-Fast qPCR Master Mix, Agilent). The primer pairs (5′-GGTTGCGTCGGAGAACATATAA-3′ and 5′-CTAAAGCTAACCTGCCACATATC-3′) and the probe (5′-FAM-TGCCCTTTCA/ZEN/GTGGGTTGAGGACAG-3′-IBFQ-3′) that amplify the viral E2 region were used. The qPCR reactions were run in AriaMx program (Agilent). Each reaction consisted of 500 nM specific primer pairs and 250 nM double-labelled probes. PCR conditions were: initial denaturation at 95°C for 10 min, then 40 cycles at 95°C for 10 min, followed by 40 cycles consisting of denaturation at 95°C for 15 s and hybridization of primers and the probe as well as DNA synthesis at 60°C for 1 min. All samples were tested in at least duplicates. Viral titres were calculated according to the standard curve. Viral copy numbers in 2 μl of a 50 μl DNA lavage extract were converted into equivalent DNA load using the formula 1 ng viral DNA = 1.2 × 10^8^ copies (http://cels.uri.edu/gsc/cndna.html). In some cases we also calculated the difference in cycle time (Ct) between the 18S rRNA gene and viral DNA (ΔCt) [24]. Fold change (2^−ΔΔ^Ct) demonstrates the relative viral DNA load in each sample as previously described [19].

### Antibody detection by ELISA

Rabbit and mouse sera were collected at the termination of the experiment. CRPV or MmuPV1 virus-like particles (VLPs) were used as the antigen for ELISA. Anti-CRPV monoclonal antibody (CRPV.1A) or anti-MmuPV1 monoclonal antibody (MPV.A4) was used as positive control and the sera of non-infected animals as negative control for the corresponding antigens. The ELISA was conducted as previously reported [29].

### In vitro neutralization assay

A rabbit cell line (RA2LT) generated in house was used for in vitro neutralization for serum collected from the CRPV infected rabbits [36]. A mouse keratinocyte cell line (K38, a generous gift from Dr Julia Reichelt, University of Newcastle, UK) was seeded at 1.5 × 10^5^ cells per well in DMEM/Ham’s F-12, with 4.5 g/l D-Glucose, 50 µM CaCl2, with L-Glutamine and Na-Pyruvate (Cedarlane), in 10% FBS with calcium depleted at 32°C. One µl of viral extract from tail papillomas was incubated with various dilutions of mouse sera (1:50–1:100 dilution) in media for 1 h at 37°C and added onto K38 cells incubated in 12-well plates at 32°C for 72 h. The cells were harvested with TRIzol reagent (Thermo Fisher).

### CRPV E1^E4 detection by RT-qPCR

Total RNA was extracted from the infected cells, and infectivity was assessed by measuring viral E1^E4 transcripts with RT-qPCR (E1^E4-forward, 5′-CATTCGAGTC ACTGCTTCTGC-3′; E1^E4-reverse, 5′-GATGCAGGTTTGTCGTTCTCC-3′; E1^E4-probe, 5′-6-carboxyfluorescein (FAM)-TGGAAAACGATAAAGCTCCTCCTCAGCC-6-carboxytetramethylrhodamine (TAMRA)-3′) as previously described [22] with a few modifications as follows: The Brilliant III RT-qPCR Master Mix (Agilent) was used for the RT-qPCR reactions. The following cycling conditions were applied: 50°C for 30 min (the reverse transcription), 95°C for 10 min, and 40 cycles of 94°C for 15 s and 60°C for 1 min. At the end of each amplification cycle, three fluorescence readings were detected. Analysis of the amplification efficiencies was performed using the REST software [19].

### Western blot analysis

Total protein from matching samples used in the RNA-seq study was isolated by homogenization in 1 × RIPA (Boston BioProducts) buffer supplemented by 1 × complete protease inhibitors (Roche). The isolated total protein was analyzed by Western blot for the expression of endogenous protein using specific antibody against APOBEC2 (Sigma-Aldrich, cat. no. SAB2500083), S100A9 (Abnova, cat. no. PAB11470) and β-tubulin (Sigma-Aldrich, cat. no. T5201).

### Immunohistochemistry and in situ hybridization analyses of infected tissues

After termination of the experiment, the animals were euthanized, and tissues of interest were fixed in 10% buffered formalin and processed to formalin-fixed paraffin-embedded (FFPE) sections as previously described [37]. Hematoxylin and eosin (H&E) analysis, in situ hybridization (ISH) and immunohistochemistry (IHC) were conducted as described in previous studies [24,37]. For IHC, an in-house anti-MmuPV1 L1 monoclonal antibody (MPV.B9) and a rabbit polyclonal antibody against MmuPV1 E4 protein (a generous gift from Dr John Doorbar) were used on FFPE sections. For ISH, a biotin labelled 2794 bp EcoRV/SacII sub-genome fragment of CRPV and 3913 bp EcoRV/BamHI sub-genomic fragment of MmuPV1 were used as in situ hybridization probes for the detection of CRPV and MmuPV1 DNA in tissues respectively [15,37]. Counterstaining for ISH was Nuclear Fast Red (American MasterTech, Inc.) and for IHC was hematoxylin (Thomas Scientific).

## Results

### Intravenous (IV) delivery of CRPV virions yields infections at skin site

**Figure 1.**
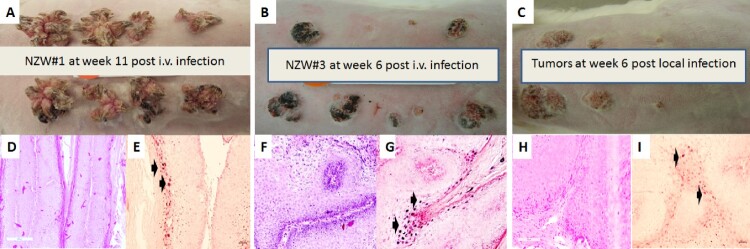
Tumour growth patterns resulting from IV infection with *CRPV virions* via marginal ear vein injection. (A) Tumour growth on one (NZW#1) of the three animals at week 11 post infection is shown. (B) one (NZW#3) of the four additional rabbits (NZW#3-6) infected by IV injection of virions equivalent to 5.5 × 10^9^ viral DNA equivalents. All developed tumours at six weeks post infection. Both tumours (A and B) exhibited an appearance similar to tumours from local skin tumours initiated with high to low dilutions of virus at week six post infection (C). (D, F) The tumours induced via marginal ear vein (IV) infection have similar morphology and histology (H&E, 20×) to those (H) initiated by local infections (H&E, 20×). Viral DNA was detected by *in situ* hybridization (ISH) in tumours induced by both intravenous (E, G) and local skin infections (I, 20×, arrows).

The rabbit model was used to test whether CRPV virus introduced into the blood stream could yield infections at pre-wounded skin sites. In a pilot study animals were infected via the marginal ear vein with CRPV virions (500 µl of the viral stock = 2.75 × 10^10^ viral DNA equivalents) (Supplementary Table 1). The amount of virus in the circulation was estimated to be 1.8 × 10^5^ copies/µl blood. Within four weeks tumours were visible at the pre-wounded sites of all animals (Supplementary Figure 1A–C). The experiment was repeated with four NZW rabbits (NZW#3-6) using five-fold fewer virions (3.6 × 10^4^/µl blood). 18 of 32 sites developed papillomas (Figure 1A–C). Representative histology of tumours from each of two experiments (Figure 1D and F, 20×) is shown. H and E staining (Figure 1G and I) similar to that seen in local skin infection (Figure 1H, 20×) is observed. Viral presence was detected by in situ hybridization (Figure 1E and G, 20× arrows) and mimics that found in rabbits with local skin infection (Figure 1I, 20× arrows).

Anti-CRPV antibodies were detected in the sera of all intravenously infected animals. (Supplementary Figure 2A). These antibodies were neutralizing in *in vitro* neutralization assays (Supplementary Figure 2B). Collectively, these findings demonstrate that CRPV circulating in the blood of rabbits can initiate infection at prewounded cutaneous sites and stimulate anti-viral immune responses.

### RNA sequencing (RNA-seq) analysis revealed similar viral transcription patterns for tumours resulting from local skin and intravenous CRPV infections

**Figure 2.**
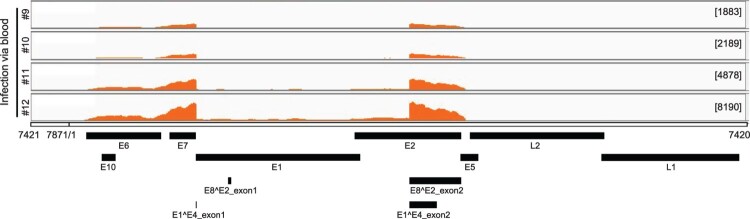
Viral RNA transcripts in four tumour lesions of four individual rabbits IV infected by CRPV virions through marginal ear vein injection. Total RNA isolated from each tumour and depleted of ribosomal RNA was analyzed by RNA-seq. By mapping the RNA-seq raw reads to the newly arranged linear CRPV genome starting from nt 7421 and ending at nt 7420 and by uploading these uniquely mapped viral RNA reads to the Integrative Genomics Viewer (IGV) program to visualize reads coverage profile along with the CRPV genome, three major coverage peaks were visualized in the E6, E7 and E1^E4 regions among all tumour tissues.

Total RNA was isolated from four tumours generated by marginal ear vein injection. The RNA was analyzed by RNA-seq. By mapping the raw reads to the newly arranged linear CRPV genome starting from nt 7421 and ending at nt 7420 using RNA sequence aligner TopHat, we obtained 18318, 24014, 62100, and 128869 viral reads for the respective tissues. These account for 0.0290%, 0.0442%, 0.0911%, and 0.1960% of total reads. By uploading the data to the Integrative Genomics Viewer (IGV) program we found three major peaks in the E6, E7 and E1^E4 regions among all tumour tissues (Figure 2).

**Figure 3.**
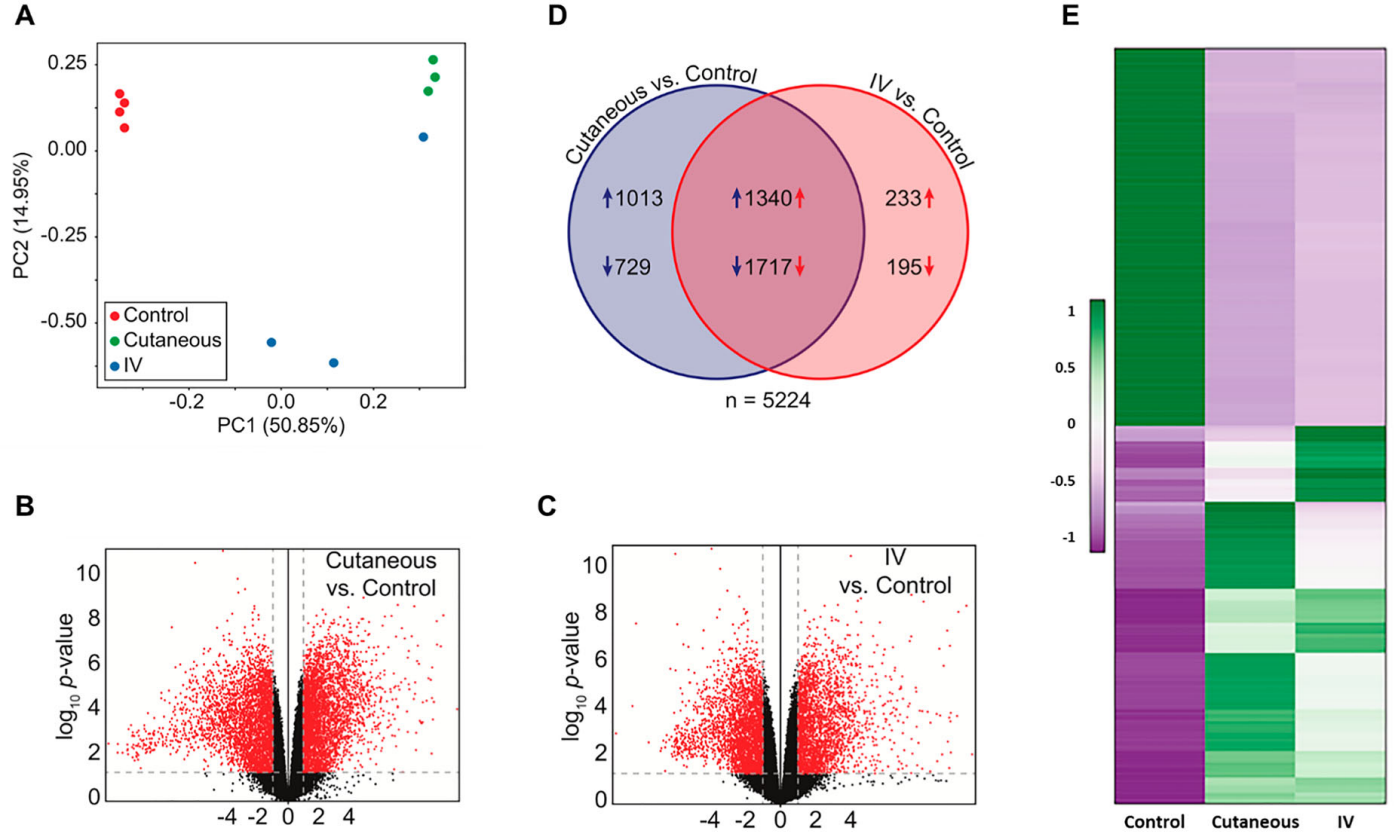
Dysregulation of the host transcriptome by CRPV infection. (A) Principle component analysis of the ten RNA-seq samples. (B-C) Volcano plots of 17742 annotated genes assayed in each contrast of our analysis. The x-axis is the log2 fold change in expression. The y-axis is *p*-value adjusted for multiple comparisons. Red dots indicate the genes with both significant differential expression and large absolute fold change relative to control; grey dots indicate those genes that do not meet these criteria. Vertical dashed lines represent fold change thresholds (absolute fold change ≥ 2.0) and horizontal dashed lines represent the significance threshold (adjusted *p* ≤ 0.05). (D) Venn diagram of all 5224 genes with differential expression (adjusted *p* ≤ 0.05 and absolute fold change ≥ 2.0) in the wart tissues induced by local skin or IV CRPV infection over the normal control skin tissues. Numbers with arrows indicate the number of genes (after all filters) up- and down-regulated in each experimental group relative to control group. (E) Heat map showing the top 100 up-regulated and top 100 down-regulated genes with significantly different expression in the tumours induced by both local skin and those induced by IV infections, relative to control. A colour scale bar represents relative gene expression level within centred rows. Unit variance scaling has been applied to rows. Both rows and columns are clustered using Euclidean distance and complete linkage.

**Figure 4.**
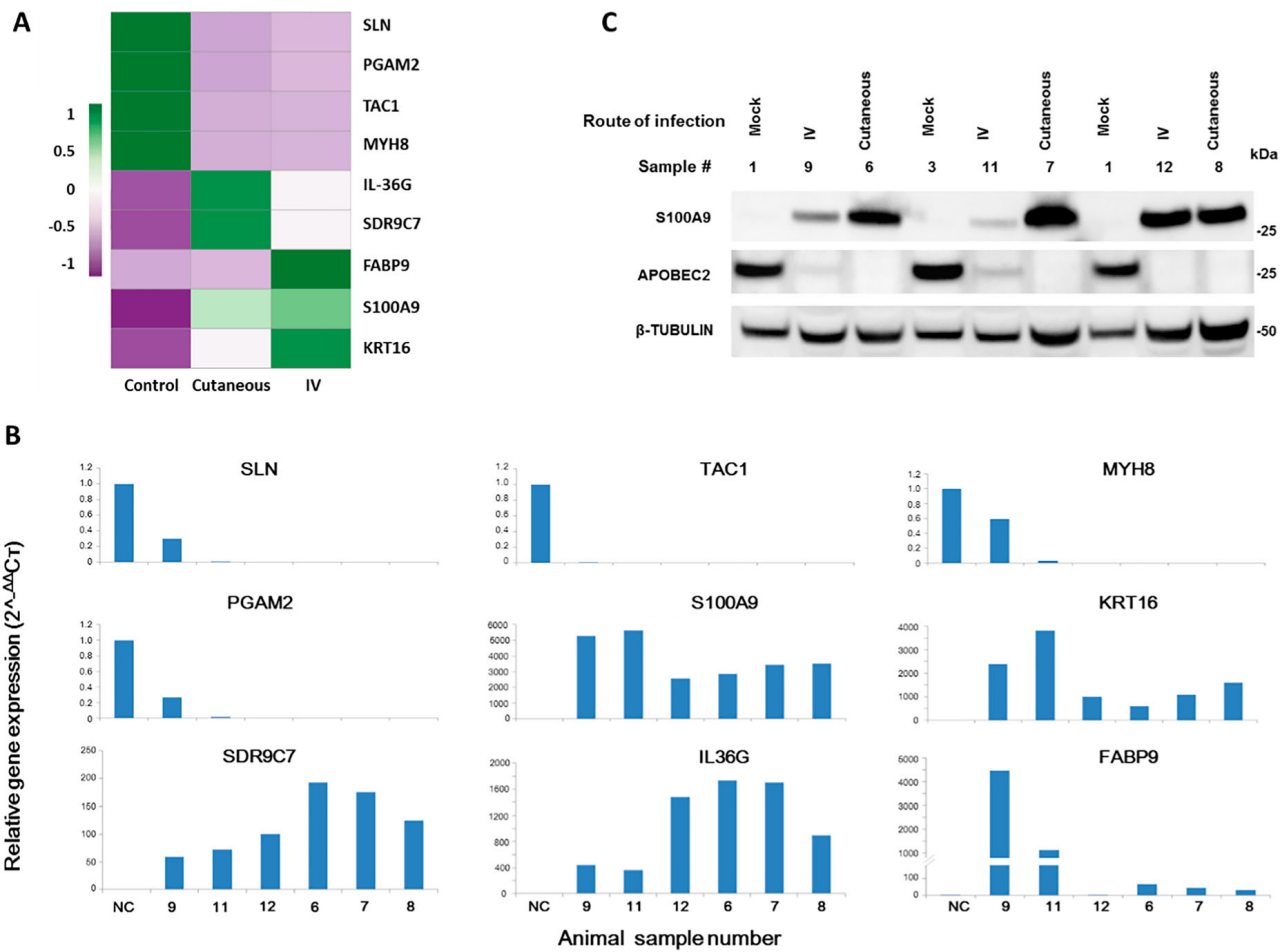
Representative host genes with differential expression in skin tumours induced by both routes of CRPV infections. (A) Heat map showing the expression of 9 selected host genes chosen based on their expression abundance and cellular functions. A colour scale bar represents relative gene expression level within centred rows. Unit variance scaling has been applied to rows. Both rows and columns are clustered using Euclidean distance and complete linkage. (B) Verification of differentially expressed rabbit genes in RNA-seq results by real-time RT-qPCR. Consistent with RNA-seq data, SLN, TAC1, MYH8, and PGAM2 were down-regulated in both CRPV blood infection (Animal #9, #11, and #12) and CRPV skin infection (Animal#6, #7, and #8) animals relative to those in normal control animals, whereas SDRC7, KRT16, S100A9, IL36G, and FABP9 were up-regulated in both CRPV skin infection and CRPV blood infection animals compared to normal controls. The Y-axis indicates relative gene expression levels calculated by 2-^ΔΔ^Cт and the X-axis indicate the different samples. NC, gene expression in the normal tissue, was set to 1 after normalization to GAPDH. (C) Western blot analysis of representative samples from normal skin and warts induced by cutaneous or intravenous (IV) CRPV infection for the expression of S100A9 and APOBEC2. Cellular β-tubulin served as a loading control.

RNA-seq data of three groups of ten tissues (four normal, three each of tumours induced by IV and local skin infection) were analyzed by Principle Component Analysis (PCA). A well-grouped dataset was found (Figure 3A). One of three tumours derived from IV infections had high virus titre by RNA-seq raw reads (Figure 2) and the altered gene expression pattern resembled that of the tumours induced by local skin infection (Figure 3A). Volcano plots of 17742 annotated genes in the rabbit genome exhibited significant differences in the host transcriptome for normal control skin group compared with the tumour groups induced either by IV or local skin infection (Figure 3B,C). Dysregulated expression is detailed in Supplemental Table 3. Approximately 3000 of 5224 genes showed similar alterations by the two routes of CRPV infections (Figure 3D). Using the thresholds of *P*≤0.05 and absolute fold change (FC) ≥2.0 of gene expression, we analyzed the top 100 up-regulated and top 100 down-regulated genes in each group (Figure 3E) by heatmap analysis and identified the most important genes with differential expression (Figure 3E), notably the genes with upregulated expression. Based on gene functions related to cell cycle, proliferation, and oncogenesis as well as their RNA abundance, we subsequently verified nine rabbit genes by real-time qPCR with significantly different expression in CRPV-induced tumours from both infection routes (Supplementary Table 3) (Figure 4A,B). Consistent with RNA-seq data, the expression of SLN, TAC1, MYH8, and PGAM2 were down-regulated, whereas SDRC7, KRT16, S100A9, IL36G, and FABP9 were up-regulated in both blood (Animal #9, #11, and #12) and skin (Animal #6, #7, and #8)-induced tumours relative to normal skin controls (Figure 4B). Consistent with RNA-seq results, western blot analysis confirmed the increased expression of S100A9 and decreased expression of APOBEC2 in tumours induced by both routes of infections (Figure 4C). These findings support the hypothesis that bloodborne and local skin papillomavirus infections have similar infectivity mechanisms.

### CRPV DNA delivered intravenously yielded infections

HPV DNA can be detected in human blood [1,38–40]. Whether this detected DNA pose a risk for infections is uncertain. CRPV DNA delivered directly to wounded sites results in tumour growth [25,26,41]. We hypothesized that CRPV DNA delivered to the blood can induce infections at permissive back skin sites. To maximize the result based on previous work (Supplementary Table 4), we inoculated 500 µg of viral DNA (estimated to be 4.6 × 10^11^ copies/µl blood) into the ear veins of two rabbits with pre-wounded back skin sites. Both rabbits grew tumours at week nine post IV infection. Histology (Supplementary Figure 3B) of one representative papilloma was similar to that of the tumours initiated by local skin infections (Figure 1H).

### Transfusion of blood containing CRPV virus yielded infections

The driving question behind our research was ‘Can transfusion of human blood containing HPV sequences result in papillomavirus infection in the recipient?’ In a small pilot study, two NZW rabbits were intravenously infected with CRPV virions (1.8 × 10^5^ copies/µl blood). Twenty-five minutes after the virus inoculation, 10 ml of blood (viral concentration was estimated to be 3 × 10^6^ copies in 10 ml blood) was drawn from each donor rabbit and infused into corresponding siblings, each with pre-wounded back sites. One recipient grew a skin tumour ten weeks post transfusion (Supplementary Figure 3C). Histology of this tumour (Supplementary Figure 3D) was similar to that shown in Figure 1K. Viral DNA was detected in the tumour by in situ hybridization (Supplementary Figure 3E, 60×).

### Athymic mice inoculated intravenously with MmuPV1 developed infections at both cutaneous and mucosal tissues

**Figure 5.**
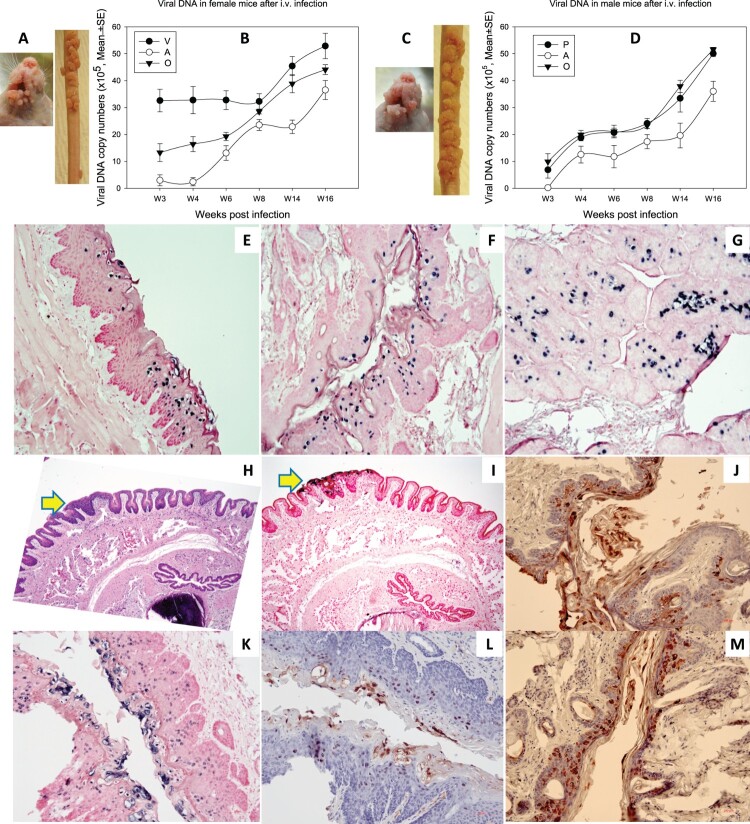
Tumour growth at cutaneous sites (muzzle and tail) and viral DNA detection at the four mucosal (vaginal, V; penile, P; anal, A; and oral, O) sites after MmuPV1 IV infection via the tail vein. Infections were introduced via the tail vein with 1 × 10^8^ viral DNA equivalent virions in eight Hsd: Nu female and six male mice that had been pre-wounded according to our standard protocol. The injection sites were treated topically with an excess of neutralizing antibody (MPV.A4) immediately post injection to neutralize any virions remaining at the site. (A, C) The animals were monitored for tumour growth at pre-wounded cutaneous sites and (B, D) for viral DNA detection by qPCR at mucosal sites. (E) tongue, (F) vaginal, (G) anal, (all 20×), were positive for viral DNA by in situ hybridization (ISH, in blue). Interestingly, dysplasia was found in the penile tissue (H&E, H, 10×, arrow) that was positive for viral DNA by ISH (I, 10×, K, 20×), viral capsid protein L1 (L, 20×), and E4 protein (J, M, 20×) by immunohistochemistry.

1 × 10^8^ viral DNA equivalent virions were injected into the tail vein of six female and six male Hsd: NU mice that had been pre-wounded at both cutaneous and mucosal sites according to our standard protocol [21,24]. The injection sites were treated topically with excess neutralizing monoclonal antibody (MPV.A4) to neutralize any virions remaining at the site (Supplementary Figure 4) [20]. All tails developed tumours at the prewounded sites (representative mouse from female and male groups respectively, Figure 5A for females and 5C for males). No tumours developed at the sites of injection indicating that MPV.A4 efficiently blocked possible skin contamination. Mucosal sites (the tongue, anus, vagina, and penis) were positive for viral DNA by qPCR (Figure 5B,D) and by in situ hybridization (ISH) (Figure 5E–G for females, I, K for male penile tissues). All tissues were positive for viral capsid L1 and E4 proteins by iommunohistochemistry. Representative figures are shown for penile tissues: L1 (Figure 5L) and E4 protein (Figure 5J,M).

### Infections were found in stomach tissues of mice infected via tail vein with MmuPV1

**Figure 6.**
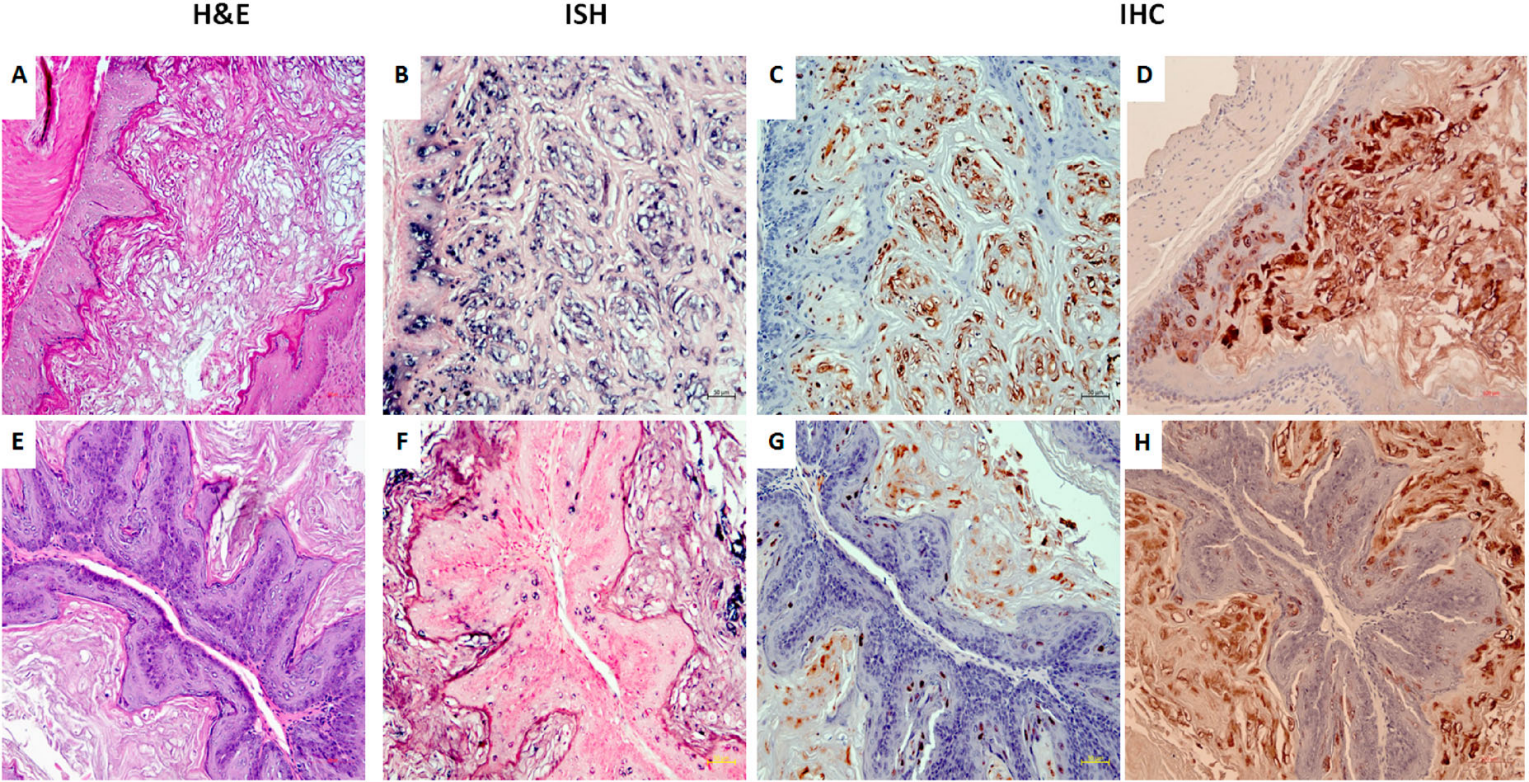
Three (two females and one male) of eleven tested mice were positive for virus infection in the stomach tissues. Representative stomach tissues were examined for histology. (A) Within the glandular stomach there is a focally extensive plaque lesion of mild to moderate hyperplasia with atypia. There is abundant hyperkeratosis (likely parakeratotic), with what appear to be crypt formation. There are occasional positive nuclei within the stratum spinosum and granulosum, with abundant positive staining of the cornified layers. There is multifocal cytoplasmic hybridization with parietal cells in the glandular stomach. (E) A small isolated (possibly pedunculated) focus of mild hyperplasia with cytologic and nuclear atypia (low grade) in the non-glandular stomach with scattered individual nuclear positive cells. These tissues were positive for viral DNA by *in situ* hybridization (ISH, B, F, 20×, in blue), viral capsid protein L1 (IHC, C, G, 20×, in red), and E4 protein (IHC, D, H, 20×, in red) by immunohistochemistry.

Papillomavirus sequences can sometimes be found in cancers of internal organs [5,6,11,12]. Therefore the organs of intravenously infected mice were examined by H&E staining, in situ hybridization and immunohistochemistry. Interestingly, the non-granular stomach tissues of three out of eleven mice were found to be positive for viral DNA, L1, and E4 proteins (Figure 6). One of the tissues showed a focally extensive plaque lesion of mild to moderate hyperplasia with atypia (Figure 6A). Abundant hyperkeratosis with what appeared to be crypt formation was observed. There were occasional positive nuclei within the stratum spinosum and granulosum with abundant positive staining of viral DNA, L1, and E4 proteins in the cornified layers. There were multifocal cytoplasmic hybridization signals within parietal cells in the glandular stomach. Two stomach tissues displayed a small isolated (possibly pedunculated) focus of mild hyperplasia with cytological and nuclear atypia (low grade) in the non-glandular stomach with scattered individual nuclear positive cells (Figure 6D). These tissues were positive for viral DNA (Figure 6B,F, 20×, in blue), viral capsid protein (Figure 6C,G, 20×, in red), and viral E4 protein (Figure 6D,H, 20×, in red). No other organs were found to be positive for MmuPV1 in these animals. This observation indicates that internal organs can become infected when the route of viral delivery is via the blood.

### Transfusion of naïve mice with blood from mice with MmuPV1 infections yielded tumours at prewounded sites

**Figure 7.**
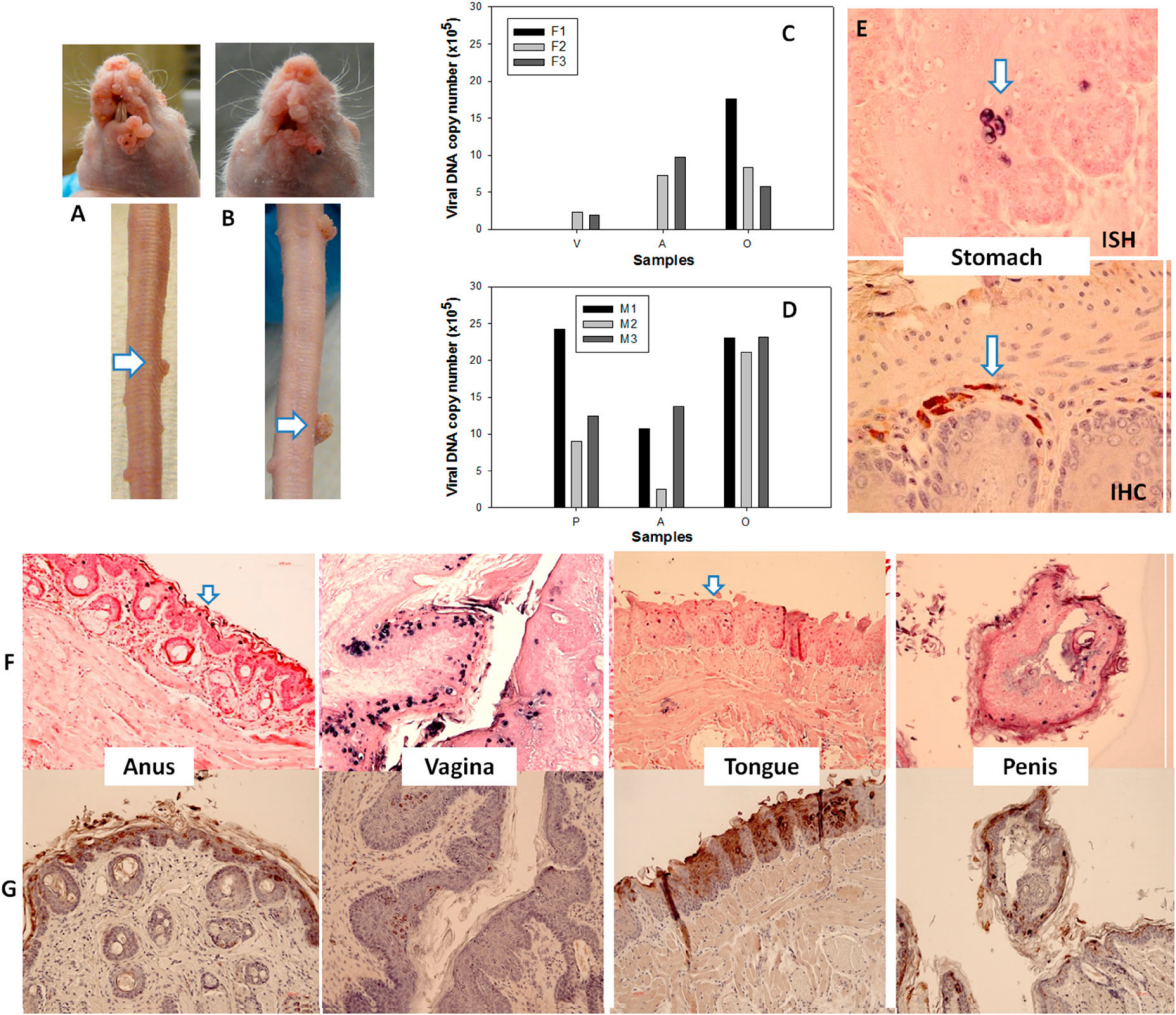
Blood of MmuPV1 infected mice with skin tumours was infectious at seven months post initial IV injection. Each naïve littermate transfused by IV injection of 0.2 mL of blood (<1 × 10^4^ viral copies) from two infected mice sacrificed seven months after initial IV MmuPV1 infection was examined weekly for tumour growth at the pre-wounded skin area. (A, B) Representative tumour growth (arrows) at the muzzle and the tail of naïve Hsd: Nu female (A) and male (B) mice at week sixteen post blood transfusion. Viral DNA was detected at the vaginal (V), anal (A) and oral (O) sites in three females (C, F1-F3) and the penile (P), anal (A) and oral (O) sites in three male (D, M1-M3) mice by qPCR. Mucosal sites of these mice (the vagina, anus, tongue, and penile) were positive for viral DNA by in situ hybridization (F, arrows, 20×, in blue). These tissues were positive for viral E4 protein (G, 20×, in red). One of the females was positive for viral DNA (ISH arrows, 60×, in blue) and viral capsid protein L1 (IHC, arrows, 60×, in red) in the stomach tissues (E).

We next examined the ability of mice with infections to transmit the disease to naïve animals via blood transfusion. Two infected mice (one male and one female) were sacrificed seven months after IV infection and 0.2 ml of blood (<1 × 10^4^ viral copies in total) from each animal was transfused via tail vein injection into three male (M1–M3) and three female (F1–F3) littermates. The injection sites were treated topically with neutralizing monoclonal antibody (MPV.A4) immediately post injection. At ten weeks post infection, all recipients were positive for viral infection and tumour growth at all of the wounded sites (Figure 7A–D). No lesions developed at the sites of injection. The tissues were positive for viral DNA (Figure 7F) and E4 protein (Figure 7G). *One stomach tissue was found to be positive for viral DNA* (ISH, 60×, in blue) *and capsid protein* (IHC, arrows, 60×, in red, Figure 7E). Thus, blood from animals with papillomavirus infections can transmit infections to naïve animals. Infection sites include the stomach.

### Viral DNA was detected in blood samples of CRPV infected rabbits and MmuPV1 infected athymic mice

To check for a viral DNA signature in the blood of infected animals, PCR and rolling circle amplification and DNA sequencing were performed to evaluate the presence of CRPV or MmuPV1 DNA in the blood of both locally and IV infected rabbits and mice. Viral DNA was detected in the whole blood of both rabbits (12/29) and mice (8/12). The highest viral titre in the rabbit and mouse blood samples was approximately 1 × 10^5^ copies/ml and 1 × 10^6^ copies/ml respectively. 12 of 31 mouse serum samples also tested positive for viral sequences but none of the rabbit sera were positive. The highest viral titre in the mouse serum sample is 2 × 10^5^ copies/ml.

## Discussion

In this study we examined the significance of papillomavirus infection in the blood. If papillomaviruses can be transmitted via the blood, patients could be at risk of HPV infections [42,43]. Blood is routinely screened for human immunodeficiency virus (HIV), hepatitis C (HCV), and hepatitis B (HBV). Procedures are being developed to detect emerging viruses such as Dengue and Zika [44]. However, there is no screening for HPVs. HPV was detected in the blood of a subset of sexually naïve children with hemophilia who had received multiple transfusions [1]. HPV sequences are sometimes found in tumours of internal organs such as the stomach, prostate, breast, colon, bladder, esophagus, and lung [3,9,11,45–49]. We asked whether these observations could be due to transmission of the virus through the blood.

All papillomaviruses are highly species specific. Therefore, a human papillomavirus cannot infect any animal [50]. Our laboratory has expertise in both the CRPV/rabbit and the MmuPV1/mouse models [15,16]. Armed with these preclinical models, we demonstrated that (1) Viral sequences could be detected in the blood of infected animals; (2) Virus introduced into the blood yielded tumours at both cutaneous and mucosal sites; (3) CRPV DNA introduced into the blood yielded papillomas at prepared skin sites; (4) Similar mechanisms are used for infections via the blood and by direct application of virus to the skin as determined by RNA-seq analysis; (5) Transfusion of blood from an animal that had received virus via intravenous infection to a naïve sibling resulted in papillomas in the transfusion recipient; (6) Virus introduced via intravenous delivery yielded infections at the stomach as well as the normally permissive sites, and (7) Blood from animals with active infections could induce infections in naïve mice when transfused into these animals.

We conducted RNA-seq analysis to compare the transcriptomes of CRPV tumours induced by local skin infection and IV infection. The patterns of viral transcription in the tumours were similar for both routes of infections. Most of the genes with significantly differential expression were common to all tumours examined by RNA-seq providing evidence that mechanisms for papillomavirus IV infection and local infection are similar. RT-qPCR and Western blotting selectively verified a subset of those genes with altered expression. For example, increased expression of S100A9 (S100 Calcium Binding Protein A9), SDR9C7 (Short Chain Dehydrogenase/Reductase Family 9C Member 7) and FABP9 (Fatty Acid Binding Protein 9) that promote cell proliferation and tumour cell migration [51,52], and KRT16 (stress keratin 16) and IL-36G (interleukin 36 gamma) that promote anti-tumour immune responses [53] was found in all CRPV-induced tumours. Decreased expression of SLN (Sarcolipin), TAC1 (Tachykinin Precursor 1), MYH8 (Myosin Heavy Chain 8), PGAM2 (Phosphoglycerate Mutase 2) and APOBEC2 (apolipoprotein-B mRNA-editing catalytic polypeptide 2) was observed in all tumours potentially leading to disruption of calcium homeostasis, signalling pathways, and sugar metabolism [54,55]. We postulate that reduced expression of APOBEC2 may prevent or reduce C-to-U editing, as described for APOBEC3 for human papillomavirus, thereby minimizing hypermutations into the CRPV genome and facilitating cancer progression [56,57].

Most individuals acquire papillomavirus infections at some point in their lifetimes [58]. Sexually transmitted infections are common in young people. Most papillomavirus infections are thought to clear spontaneously, but the process often occurs over a considerable time period [59]. It is possible that virus from acute or latent infections makes its way, in some yet to be determined manner, into the blood stream [60]. Our results from animal studies would support this. Because we demonstrated in this study that both viral DNA and virions injected into the blood stream could induce tumours, we did not treat transfusion samples with an agent such as Benzonase to remove externally bound DNA or RNA prior to transfusion. Transfusion of this blood containing either virions or viral DNA into another individual, especially one with a compromised immune system, could pose a risk of infection to the recipient. These infections might manifest not only in the genital sites normally associated with the virus but also in distant vital organs, as shown in this paper where the virus was found in the stomachs of four different animals.

The link between a blood transfusion and an infection or cancer that manifests much later in life would not be easy to detect in hindsight. In view of the results presented here, it would seem prudent to conduct studies on human blood samples to ascertain the need for routine screening to assure safety of the blood supply.

## Supplementary Material

Supplemental Material

## Abbreviations

Abbreviations: HPV, Human Papillomavirus; CRPV, The Cottontail Rabbit Papillomavirus; MmuPV1, The Mouse Papillomavirus; NZW, New Zealand White; IV, Intravenous; PBMCs, Peripheral Blood Mononuclear Cells; PCA, Principle Component Analysis;

Definitions: Local papillomavirus infection: An infection initiated by the direct application of virus or viral DNA to the site of infection; Intravenous (IV) papillomavirus infection: An infection resulting from blood-borne delivery of virus or viral DNA to the site of infection

## References

[1] Bodaghi S, Wood LV, Roby G, et al. Could human papillomaviruses be spread through blood? J Clin Microbiol. 2005;43:5428–5434. doi:10.1128/JCM.43.11.5428-5434.2005. 16272465 PMC1287818

[2] Taberna M, Mena M, Pavon MA, et al. Human papillomavirus-related oropharyngeal cancer. Ann Oncol. 2017;28:2386–2398. doi:10.1093/annonc/mdx304. 28633362

[3] Agalliu I, Chen Z, Wang T, et al. Oral Alpha, Beta and Gamma HPV types and risk of incident esophageal cancer. Cancer Epidemiol Biomarkers Prev. 2018. doi:10.1158/1055-9965.EPI-18-0287. PMC6170688 30087123

[4] Shikova E, Ivanova Z, Alexandrova D, et al. Human papillomavirus prevalence in lung carcinomas in Bulgaria. Microbiol Immunol. 2017;61:427–432. doi:10.1111/1348-0421.12535. 28881043

[5] Bodaghi S, Yamanegi K, Xiao SY, et al. Colorectal papillomavirus infection in patients with colorectal cancer. Clin Cancer Res. 2005;11:2862–2867. doi:10.1158/1078-0432.CCR-04-1680. 15837733 PMC1479314

[6] Baandrup L, Thomsen LT, Olesen TB, et al. The prevalence of human papillomavirus in colorectal adenomas and adenocarcinomas: a systematic review and meta-analysis. Eur J Cancer. 2014;50:1446–1461. doi:S0959-8049(14)00092-6 [pii]; 10.1016/j.ejca.2014.01.019 [doi] doi: 10.1016/j.ejca.2014.01.019 24560489

[7] Tachezy R, Hrbacek J, Heracek J, et al. HPV persistence and its oncogenic role in prostate tumors. J Med Virol. 2012;84:1636–1645. doi:10.1002/jmv.23367. 22930513

[8] Glenn WK, Ngan CC, Amos TG, et al. High risk human papilloma viruses (HPVs) are present in benign prostate tissues before development of HPV associated prostate cancer. Infect Agent Cancer. 2017;12:46. doi:10.1186/s13027-017-0157-2. 28811834 PMC5553674

[9] Malhone C, Longatto-Filho A, Filassi JR. Is human papilloma virus associated with breast cancer? A review of the molecular evidence. Acta Cytol. 2018;62:166–177. doi:10.1159/000487700. 29694946

[10] ElAmrani A, Gheit T, Benhessou M, et al. Prevalence of mucosal and cutaneous human papillomavirus in Moroccan breast cancer. Papillomavirus Res. 2018;5:150–155. doi:10.1016/j.pvr.2018.04.003. 29660489 PMC5909064

[11] Mirzaei H, Goudarzi H, Eslami G, et al. Role of viruses in gastrointestinal cancer. J Cell Physiol. 2018;233:4000–4014. doi:10.1002/jcp.26194. 28926109

[12] Zeng ZM, Luo FF, Zou LX, et al. Human papillomavirus as a potential risk factor for gastric cancer: a meta-analysis of 1,917 cases. Onco Targets Ther. 2016;9:7105–7114. doi:10.2147/OTT.S115053. 27895502 PMC5119622

[13] Martinez GG, Troconis JN. [Natural history of the infection for human papillomavirus: an actualization]. Invest Clin. 2014;55:82–91. 24758104

[14] Doorbar J. Model systems of human papillomavirus-associated disease. J Pathol. 2016;238:166–179. doi:10.1002/path.4656. 26456009

[15] Cladel NM, Peng X, Christensen N, et al. The rabbit papillomavirus model: a valuable tool to study viral-host interactions. Philos Trans R Soc Lond B Biol Sci. 2019;374:20180294. doi:10.1098/rstb.2018.0294. 30955485 PMC6501911

[16] Hu J, Cladel NM, Budgeon LR, et al. The mouse papillomavirus infection model. Viruses. 2017;9; doi:10.3390/v9090246. PMC5618012 28867783

[17] Uberoi A, Lambert PF. Rodent papillomaviruses. Viruses. 2017;9; doi:10.3390/v9120362. PMC5744137 29186900

[18] Brandsma JL. The cottontail rabbit papillomavirus model of high-risk HPV-induced disease. Methods Mol. Med. 2005;119:217–235. 16350405 10.1385/1-59259-982-6:217

[19] Cladel NM, Budgeon LR, Balogh KK, et al. Mouse papillomavirus infection persists in mucosal tissues of an immunocompetent mouse strain and progresses to cancer. Sci Rep. 2017;7:16932. doi:10.1038/s41598-017-17089-4. 29208932 PMC5717108

[20] Cladel NM, Budgeon LR, Cooper TK, et al. Mouse papillomavirus infections spread to cutaneous sites with progression to malignancy. J Gen Virol. 2017. doi:10.1099/jgv.0.000926. PMC5845569 28942760

[21] Cladel NM, Budgeon LR, Cooper TK, et al. Secondary infections, expanded tissue tropism, and evidence for malignant potential in immunocompromised mice infected with Mus musculus papillomavirus 1 DNA and virus. J. Virol. 2013;87:9391–9395. doi:JVI.00777-13 [pii]; 10.1128/JVI.00777-13 [doi] doi: 10.1128/JVI.00777-13 23785210 PMC3754027

[22] Hu J, Budgeon LR, Cladel NM, et al. Detection of L1, infectious virions and anti-L1 antibody in domestic rabbits infected with cottontail rabbit papillomavirus. J. Gen. Virol. 2007;88:3286–3293. doi: 10.1099/vir.0.82879-0 18024897

[23] Cladel NM, Budgeon LR, Balogh KK, et al. Mouse papillomavirus MmuPV1 infects oral mucosa and preferentially targets the base of the tongue. Virology. 2016;488:73–80. doi:10.1016/j.virol.2015.10.030. 26609937 PMC4744521

[24] Hu J, Budgeon LR, Cladel NM, et al. Tracking vaginal, anal and oral infection in a mouse papillomavirus infection model. J Gen Virol. 2015;96:3554–3565. doi:10.1099/jgv.0.000295. 26399579 PMC4804763

[25] Cladel NM, Hu J, Balogh K, et al. Wounding prior to challenge substantially improves infectivity of cottontail rabbit papillomavirus and allows for standardization of infection. J Virol Methods. 2008;148:34–39. doi: 10.1016/j.jviromet.2007.10.005 18061687 PMC2278115

[26] Kreider JW, Cladel NM, Patrick SD, et al. High efficiency induction of papillomas in vivo using recombinant cottontail rabbit papillomavirus DNA. J Virol Methods. 1995;55:233–244. doi: 10.1016/0166-0934(95)00062-Y 8537461

[27] Xiao W, Brandsma JL. High efficiency, long-term clinical expression of cottontail rabbit papillomavirus (CRPV) DNA in rabbit skin following particle-mediated DNA transfer. Nucleic Acids Res. 1996;24:2620–2622. doi: 10.1093/nar/24.13.2620 8692707 PMC145979

[28] Cladel NM, Hu J, Balogh KK, et al. Differences in methodology, but not differences in viral strain, account for variable experimental outcomes in laboratories utilizing the cottontail rabbit papillomavirus model. J Virol Methods. 2010;165:36–41. doi: 10.1016/j.jviromet.2009.12.014 20036285 PMC2845176

[29] Hu J, Budgeon LR, Balogh KK, et al. Long-peptide therapeutic vaccination against CRPV-induced papillomas in HLA-A2.1 transgenic rabbits. Trials Vaccinol. 2014;3:134–142. doi:10.1016/j.trivac.2014.06.002. 25243025 PMC4165355

[30] Xue XY, Majerciak V, Uberoi A, et al. The full transcription map of mouse papillomavirus type 1 (MmuPV1) in mouse wart tissues. PLoS Pathog. 2017;13:e1006715. doi:10.1371/journal.ppat.1006715. 29176795 PMC5720830

[31] Bolger AM, Lohse M, Usadel B. Trimmomatic: a flexible trimmer for Illumina sequence data. Bioinformatics. 2014;30:2114–2120. doi:10.1093/bioinformatics/btu170. 24695404 PMC4103590

[32] Dobin A, Davis CA, Schlesinger F, et al. STAR: ultrafast universal RNA-seq aligner. Bioinformatics. 2013;29:15–21. doi:10.1093/bioinformatics/bts635. 23104886 PMC3530905

[33] Li B, Dewey CN. RSEM: accurate transcript quantification from RNA-Seq data with or without a reference genome. BMC Bioinformatics. 2011;12:323. doi:10.1186/1471-2105-12-323. 21816040 PMC3163565

[34] Phipson B, Lee S, Majewski IJ, et al. Robust hyperparameter estimation protects against hypervariable genes and improves power to detect differential expression. Ann Appl Stat. 2016;10:946–963. doi:10.1214/16-AOAS920. 28367255 PMC5373812

[35] Metsalu T, Vilo J. Clustvis: a web tool for visualizing clustering of multivariate data using principal component analysis and heatmap. Nucleic Acids Res. 2015;43:W566–W570. doi:10.1093/nar/gkv468. 25969447 PMC4489295

[36] Hu J, Cladel N, Balogh K, et al. Mucosally delivered peptides prime strong immunity in HLA-A2.1 transgenic rabbits. Vaccine. 2010;28:3706–3713. doi:10.1016/j.vaccine.2010.03.015. 20332046 PMC2879011

[37] Cladel NM, Budgeon LR, Balogh KK, et al. A novel pre-clinical murine model to study the life cycle and progression of cervical and anal papillomavirus infections. PLoS One. 2015;10:e0120128. doi:10.1371/journal.pone.0120128. 25803616 PMC4372414

[38] Cocuzza CE, Martinelli M, Sina F, et al. Human papillomavirus DNA detection in plasma and cervical samples of women with a recent history of low grade or precancerous cervical dysplasia. PLoS One. 2017;12:e0188592. doi:10.1371/journal.pone.0188592. 29182627 PMC5705130

[39] Jeannot E, Becette V, Campitelli M, et al. Circulating human papillomavirus DNA detected using droplet digital PCR in the serum of patients diagnosed with early stage human papillomavirus-associated invasive carcinoma. J Pathol Clin Res. 2016;2:201–209. doi:10.1002/cjp2.47. 27917295 PMC5129558

[40] Chen AC, Keleher A, Kedda MA, et al. Human papillomavirus DNA detected in peripheral blood samples from healthy Australian male blood donors. J Med Virol. 2009;81:1792–1796. doi:10.1002/jmv.21592. 19697401

[41] Hu J, Cladel NM, Pickel MD, et al. Amino Acid residues in the carboxy-terminal region of cottontail rabbit papillomavirus e6 influence spontaneous regression of cutaneous papillomas. J. Virol. 2002;76:11801–11808. doi: 10.1128/JVI.76.23.11801-11808.2002 12414922 PMC136889

[42] Laffort C, Le Deist F, Favre M, et al. Severe cutaneous papillomavirus disease after haemopoietic stem-cell transplantation in patients with severe combined immune deficiency caused by common gammac cytokine receptor subunit or JAK-3 deficiency. Lancet. 2004;363:2051–2054. doi: 10.1016/S0140-6736(04)16457-X 15207958

[43] Shanis D, Anandi P, Grant C, et al. Risks factors and timing of genital human papillomavirus (HPV) infection in female stem cell transplant survivors: a longitudinal study. Bone Marrow Transplant. 2018;53:78–83. doi:10.1038/bmt.2017.210. 29035398

[44] Stramer SL. Current perspectives in transfusion-transmitted infectious diseases: emerging and re-emerging infections. ISBT Sci Ser. 2014;9:30–36. doi:10.1111/voxs.12070. 25210533 PMC4142007

[45] Russo GI, Calogero AE, Condorelli RA, et al. Human papillomavirus and risk of prostate cancer: a systematic review and meta-analysis. Aging Male. 2018: 1–7. doi:10.1080/13685538.2018.1455178. 29571270

[46] Akhtar N, Bansal JG. Risk factors of lung cancer in nonsmoker. Curr Probl Cancer. 2017;41:328–339. doi:10.1016/j.currproblcancer.2017.07.002. 28823540

[47] Salyakina D, Tsinoremas NF. Viral expression associated with gastrointestinal adenocarcinomas in TCGA high-throughput sequencing data. Hum Genomics. 2013;7:23. doi:10.1186/1479-7364-7-23. 24279398 PMC3906926

[48] Damin DC, Caetano MB, Rosito MA, et al. Evidence for an association of human papillomavirus infection and colorectal cancer. Eur J Surg Oncol. 2007;33:569–574. doi:10.1016/j.ejso.2007.01.014. 17321098

[49] Shigehara K, Sasagawa T, Namiki M. Human papillomavirus infection and pathogenesis in urothelial cells: a mini-review. J Infect Chemother. 2014;20:741–747. doi:10.1016/j.jiac.2014.08.033. 25271131

[50] Campo MS. Animal models of papillomavirus pathogenesis. Virus Res. 2002;89:249–261. doi: 10.1016/S0168-1702(02)00193-4 12445664

[51] Zha H, Li X, Sun H, et al. S100a9 promotes the proliferation and migration of cervical cancer cells by inducing epithelialmesenchymal transition and activating the Wnt/betacatenin pathway. Int J Oncol. 2019;55:35–44. doi:10.3892/ijo.2019.4793. 31059008 PMC6561615

[52] Tang S, Gao L, Bi Q, et al. SDR9C7 promotes lymph node metastases in patients with esophageal squamous cell carcinoma. PLoS One. 2013;8:e52184. doi:10.1371/journal.pone.0052184. 23341893 PMC3544840

[53] Zhussupbekova S, Sinha R, Kuo P, et al. A mouse model of hyperproliferative human epithelium validated by keratin profiling shows an aberrant cytoskeletal response to injury. EBioMedicine. 2016;9:314–323. doi:10.1016/j.ebiom.2016.06.011. 27333029 PMC4972546

[54] Liu MY, Zhang H, Hu YJ, et al. Identification of key genes associated with cervical cancer by comprehensive analysis of transcriptome microarray and methylation microarray. Oncol Lett. 2016;12:473–478. doi:10.3892/ol.2016.4658. 27347167 PMC4907103

[55] Xu Y, Li F, Lv L, et al. Oxidative stress activates SIRT2 to deacetylate and stimulate phosphoglycerate mutase. Cancer Res. 2014;74:3630–3642. doi:10.1158/0008-5472.CAN-13-3615. 24786789 PMC4303242

[56] Vartanian JP, Guetard D, Henry M, et al. Evidence for editing of human papillomavirus DNA by APOBEC3 in benign and precancerous lesions. Science. 2008;320:230–233. doi:10.1126/science.1153201. 18403710

[57] Bierkens M, Krijgsman O, Wilting SM, et al. Focal aberrations indicate EYA2 and hsa-miR-375 as oncogene and tumor suppressor in cervical carcinogenesis. Genes Chromosomes Cancer. 2013;52:56–68. doi:10.1002/gcc.22006. 22987659

[58] Gravitt PE, Winer RL. Natural history of HPV infection across the lifespan: role of viral latency. Viruses. 2017;9; doi:10.3390/v9100267. PMC5691619 28934151

[59] Shew ML, Ermel AC, Tong Y, et al. Episodic detection of human papillomavirus within a longitudinal cohort of young women. J Med Virol. 2015;87:2122–2129. doi:10.1002/jmv.24284. 26112742

[60] Moustafa A, Xie C, Kirkness E, et al. The blood DNA virome in 8,000 humans. PLoS Pathog. 2017;13:e1006292), doi:10.1371/journal.ppat.1006292. 28328962 PMC5378407

