# Supplementary material for: Papillomavirus can be transmitted through the blood and produce infections in blood recipients: Evidence from two animal models
Source: Emerg Microbes Infect. 2019 Jul 25;8(1):1108–21. doi: 10.1080/22221751.2019.1637072 (PMC6713970; doi:10.1080/22221751.2019.1637072)

**Supplementary Figure 1.** **Tumor growth patterns resulting from IV infection with *CRPV virions* via marginal ear vein injection**. **(A-C)** Two NZW rabbits (NZW#1-2) (A, B) and one HLA-A2.1 transgenic rabbit (A2) (C) infected by IV injection of virions equivalent to 2.75×10^10^ copies of viral DNA displayed tumors that mimic those by local skin infections in our previous studies^22^.


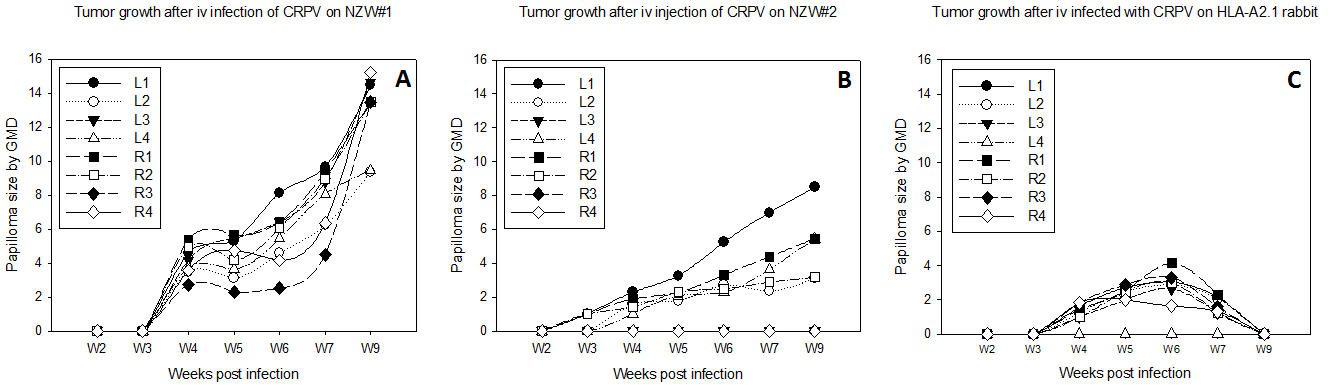


**Supplementary Figure 2.** Neutralizing antibodies were induced in the intravenously infected rabbits. Positive control was anti-rabbit L1 (CRPV.1A) and the negative control was serum of a non-infected rabbit for both ELISA and in vitro neutralization analyses. All seven IV infected animals generated specific anti-CRPV antibodies. (A) The antibody titer in ELISA assay was not correlated with the tumor size. (B) These anti-CRPV antibodies were neutralizing by the in vitro neutralizing assay.
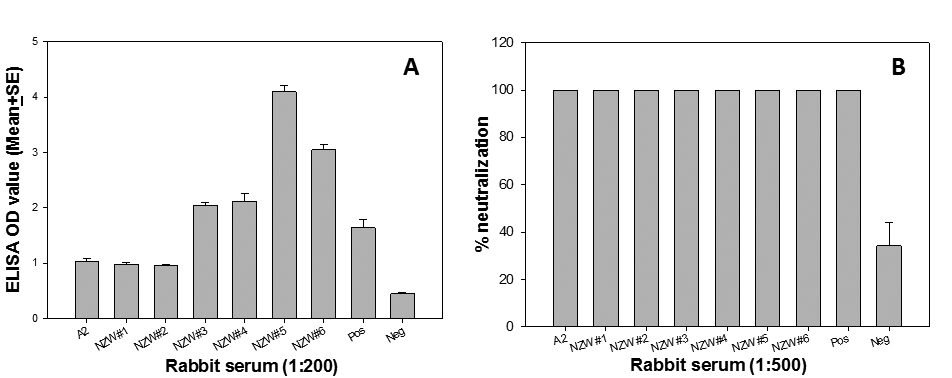


**Supplementary Figure 3. Tumor growth by IV infection with *CRPV DNA* via marginal ear vein. (A-B)** Three tumors were detected at pre-wounded back skin sites on one rabbit at week nine post intravenously infected with CRPV DNA via the marginal ear vein injection. **(B)** There are multifocal dense leukocytic infiltrates at the dermal-epidermal junctions within the tumor mass, predominantly macrophages with fewer lymphocytes and rare neutrophils in a representative tumor (H&E, 20×). Epithelial polarization and differentiation are maintained from basement membrane to the surface, with occasional mitoses and mild nuclear atypia. There are occasional koilocytosis and scattered individual apoptotic keratinocytes within the epithelium. There is no invasion present in examined tissues. This tumor has similar histology to those initiated by local skin infection as shown in Fig. 1 K. **(C)** A tumor was detected at one recipient rabbit’s back pre-wounded skin site at week ten after receiving a blood (approximately 3×10^6^ copies in 10ml blood) transfusion from a rabbit that had been infected with CRPV virions (1.8×10^5^ copies/µl) via the ear vein for 30 minutes. **(D)** The tumor has exophytic polypoid cutaneous warty masses similar to Fig. 4B, but with minimal inflammation. There is a mixture of ortho- and parakeratotic debris on the surface of the masses. **(E)** Viral DNA was detected in the tumor by in situ hybridization (ISH, 60×).


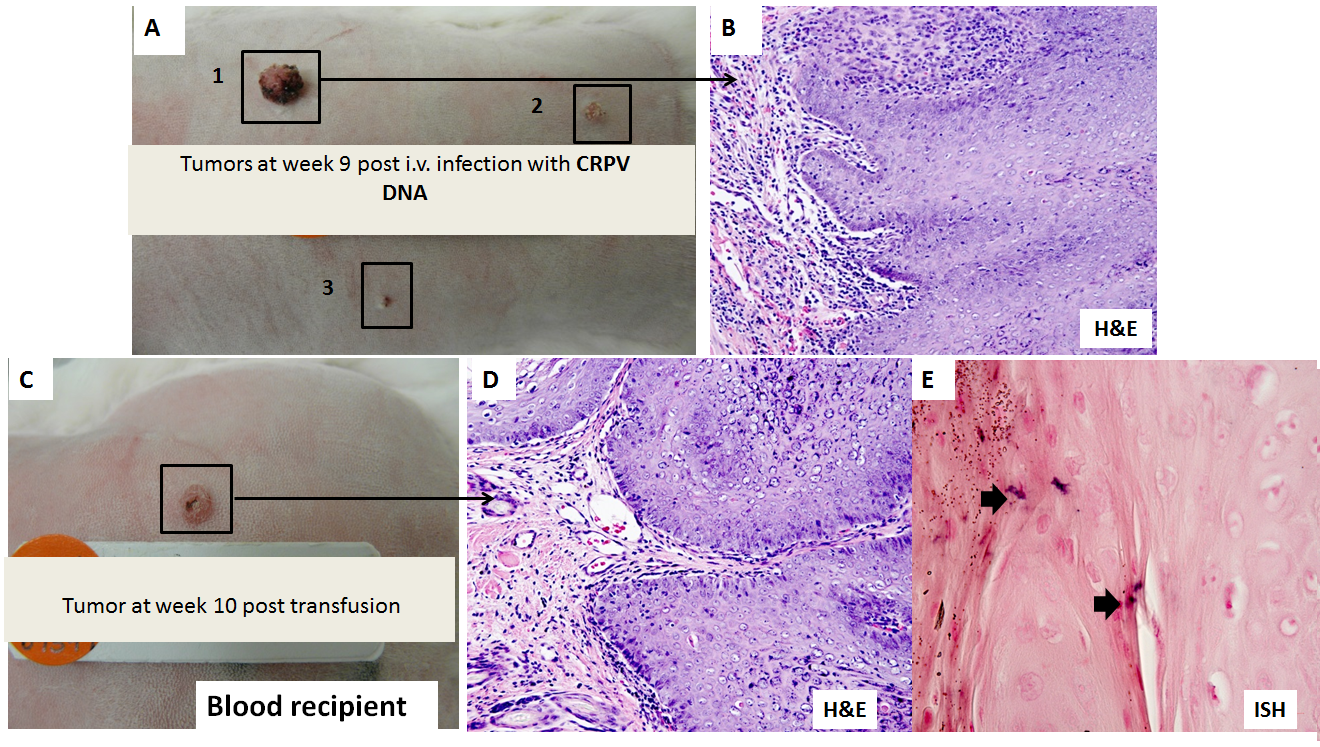


**Supplementary Figure 4.** HSD: Nu nude mice inoculated with anti-MmuPV1neutralizing antibody (MPV.A4) or a control monoclonal antibody (H11.B2 ) via i.p. at 24 hours before MmuPV1 infection at both cutaneous and mucosal sites. Viral infections can be detected at both cutaneous and mucosal by either visualization (B) or Q-PCR analysis (C) in the control group (H11.B2) but not in the test group (MPV.A4) (A, C) suggesting the neutralizing antibody can completely block viral infections in all the tested tissues.


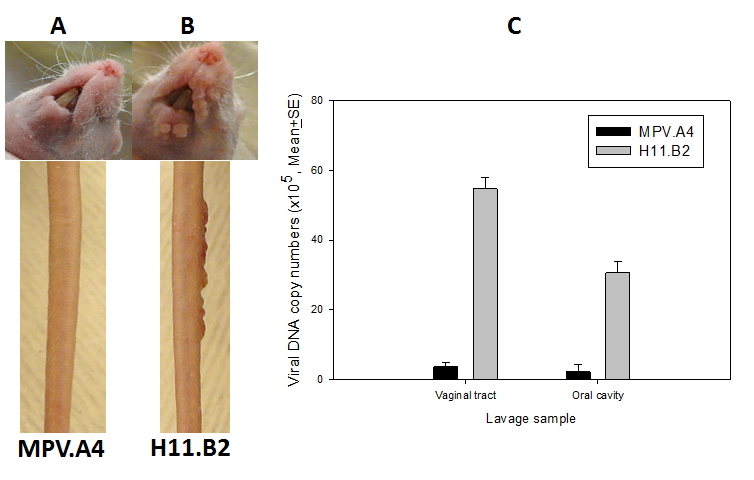

Supplement: Supplemental Material [file TEMI_A_1637072_SM1530.zip › EMISupplementary_materials4_4_19.docx]
