# Supplementary material for: Papillomavirus can be transmitted through the blood and produce infections in blood recipients: Evidence from two animal models
Source: Emerg Microbes Infect. 2019 Jul 25;8(1):1108–21. doi: 10.1080/22221751.2019.1637072 (PMC6713970; doi:10.1080/22221751.2019.1637072)
Supplement: Supplemental Material [file TEMI_A_1637072_SM1530.zip › Supplementary_Table_1.docx]

**Supplementary Table 1. Summary of CRPV virion titration at NZW domestic rabbit back sites**

| Viral titration | Viral genome equivalents | Papilloma appearance (tumor sites/infected skin sites) | |
| --- | --- | --- | --- |
|  |  | Without pre-wounding | With pre-wounding |
| 10^-1^ | 2.75×10_8_ | ND | 12/12 |
| 10^-2^ | 2.75×10_7_ | 5/5 | 33/33 |
| 10^-3^ | 2.75×10_6_ | 2/8 | 24/24* |
| 10^-4^ | 2.75×10_5_ | 0/16 | 28/36* |
| 10^-5^ | 2.75×10_4_ | 0/8 | 6/36 |
| 10^-6^ | 2.75×10_3_ | 0/8 | 1/20 |
| 10^-7^ | 2.75×10_2_ | ND | 0/12 |

*P<0.01 with pre-wounding vs. without pre-wounding, Fisher’s exact test
