# Supplementary material for: Papillomavirus can be transmitted through the blood and produce infections in blood recipients: Evidence from two animal models
Source: Emerg Microbes Infect. 2019 Jul 25;8(1):1108–21. doi: 10.1080/22221751.2019.1637072 (PMC6713970; doi:10.1080/22221751.2019.1637072)
Supplement: Supplemental Material [file TEMI_A_1637072_SM1530.zip › Supplementary_Table_2.docx]

**Supplementary Table 2. Primers and probes for RT-qPCR of rabbit gene transcripts**

| Gene names | Primers/probes | Sequence (From 5’- to 3’-) |
| --- | --- | --- |
| GAPDH | Forward primer | GACCACTTTGTGAAGCTCATTTC |
|  | Backward primer | GTGGTTTGAGGGCTCTTACTC |
|  | Taqman probe | ATTTGGCTACAGCAACAGGGTGGT |
| IL36G | Forward primer | GTTGGGAAGCTCTCCGATTT |
|  | Backward primer | CGGATACTTGCACGGCATAA |
|  | Taqman probe | TGACAGTTCCAAGGAGTAGCAACGC |
| SLN | Forward primer | CGTGTGTCCTTGACCTTCTT |
|  | Backward primer | GTGGATCGCTCCATTCTCAG |
|  | Taqman probe | AAGCCTGCCACAAGTTCTCACTGA |
| MYH8 | Forward primer | CTTGGACATTGCAGGCTTTG |
|  | Backward primer | GTTTCTCGTTGGTGAAGTTGATG |
|  | Taqman probe | TGATTTCAACAGCCTGGAGCAGCT |
| FABP9 | Forward primer | CAGAACAGAGAGTCCTTTCAGG |
|  | Backward primer | GAGCCACTGTCCAATGTTACT |
|  | Taqman probe | ACAGCAGACAACCGGAAAGTGAAGA |
| PGAM2 | Forward primer | CCCTTCTGGAACGAGGAGAT |
|  | Backward primer | GGCAGATTCAGCTCCATGAT |
|  | Taqman probe | CAAACACCTGGAAGGGATGTCGGA |
| KRT16 | Forward primer | GAGCTACGCAGGGTGTTC |
|  | Backward primer | GTAGCGGCCTTTGGTCTC |
|  | Taqman probe | AGGCTGTTCTCCAGGGATGCTTTC |
| SDR9C7 | Forward primer | GGCTACTGTGTCTCCAAGTTT |
|  | Backward primer | CCCAGGCTCAATGATGCTAA |
|  | Taqman probe | CCTTCTCTGACAGCATCAGGCGTG |

Note: Taqman assays of TAC1 (Thermo Fisher 5'-FAM,3'-MGB, predesigned, premixed, Assay ID: Oc03399333_m1) and S100A9 (Thermo Fisher 5'-FAM,3'-MGB, predesigned, premixed, Assay ID: Oc03395775_m1) were ordered from Thermo Fisher.
