# Supplementary material for: Papillomavirus can be transmitted through the blood and produce infections in blood recipients: Evidence from two animal models
Source: Emerg Microbes Infect. 2019 Jul 25;8(1):1108–21. doi: 10.1080/22221751.2019.1637072 (PMC6713970; doi:10.1080/22221751.2019.1637072)
Supplement: Supplemental Material [file TEMI_A_1637072_SM1530.zip › Supplementary_Table_4.docx]

**Supplementary Table 4. Summary of CRPV DNA titration at NZW domestic rabbit back sites**

| Viral DNA (µg) | Viral genome equivalents | Papilloma appearance (tumor sites/infected skin sites) | | | |
| --- | --- | --- | --- | --- | --- |
|  |  | Without pre-wounding | | With pre-wounding | |
| 20 | 2.6×10^12^ | ND | 28/28 | |  |
| 10 | 1.3×10_12_ | 3/5 | 4/5 | |  |
| 5 | 6.5×10_11_ | 5/8 | 11/11 | |  |
| 1 | 1.3×10_11_ | 4/5 | 11/11 | |  |
| 0.2 | 2.6×10_10_ | 2/5 | 11/11* | |  |
| 0.1 | 1.3×10_10_ | 2/5 | 3/3 | |  |
| 0.04 | 5.2×10_9_ | 3/5 | 9/11 | |  |
| 0.02 | 2.6×10_9_ | 0/3 | 1/3 | |  |
| 0.01 | 1.3×10_9_ | 1/3 | 1/3 | |  |

*P<0.05 with pre-wounding vs. without pre-wounding, Fisher’s exact test
